# Bipolar disorder: an association between body mass index and cingulate gyrus fractional anisotropy not mediated by systemic inflammation

**DOI:** 10.47626/2237-6089-2020-0132

**Published:** 2022-09-13

**Authors:** Ramiro Reckziegel, Francisco Diego Rabelo-da-Ponte, Jacson Gabriel Feiten, Isadora Bosini Remus, Pedro Domingues Goi, Miréia Fortes Vianna-Sulzbach, Raffael Massuda, Danielle Macedo, David de Lucena, Letícia Sanguinetti Czepielewski, Clarissa Severino Gama

**Affiliations:** 1 Laboratório de Psiquiatria Molecular Hospital de Clínicas de Porto Alegre Porto Alegre RS Brazil Laboratório de Psiquiatria Molecular, Hospital de Clínicas de Porto Alegre (HCPA), Porto Alegre, RS, Brazil.; 2 Programa de Pós-Graduação em Psiquiatria e Ciências do Comportamento Departamento de Psiquiatria e Medicina Legal Universidade Federal do Rio Grande do Sul Porto Alegre RS Brazil Programa de Pós-Graduação em Psiquiatria e Ciências do Comportamento, Departamento de Psiquiatria e Medicina Legal, Universidade Federal do Rio Grande do Sul (UFRGS), Porto Alegre, RS, Brazil.; 3 Departamento de Psiquiatria Universidade Federal do Paraná Curitiba PR Brazil Departamento de Psiquiatria, Universidade Federal do Paraná, Curitiba, PR, Brazil.; 4 Laboratório de Psiquiatria Translacional Núcleo de Pesquisa e Desenvolvimento de Medicações Universidade Federal do Ceará Fortaleza CE Brazil Laboratório de Psiquiatria Translacional, Núcleo de Pesquisa e Desenvolvimento de Medicações, Universidade Federal do Ceará, Fortaleza, CE, Brazil.

**Keywords:** Bipolar disorder, obesity, white matter, diffusion tensor imaging

## Abstract

**Objective:**

To investigate associations between body mass index (BMI), white matter fractional anisotropy (FA), and C-reactive protein (CRP) in a group of individuals with bipolar disorder (BD) during euthymia and compare them with a control group of healthy subjects (CTR).

**Methods:**

The sample consisted of 101 individuals (BD n = 35 and CTR n = 66). Regions of interest (ROI) were defined using a machine learning approach. For each ROI, a regression model tested the association between FA and BMI, controlling for covariates. Peripheral CRP levels were assayed, correlated with BMI, and included in a mediation analysis.

**Results:**

BMI predicted the FA of the right cingulate gyrus in BD (AdjR^2^ = 0.312 F_(3)_ = 5.537 p = 0.004; β = -0.340 p = 0.034), while there was no association in CTR. There was an interaction effect between BMI and BD diagnosis (F_(5)_ = 3.5857 p = 0.012; Fchange = 0.227 AdjR^2^ = 0.093; β = -1.093, p = 0.048). Furthermore, there was a positive correlation between BMI and CRP in both groups (AdjR^2^ = 0.170 F_(3)_ = 7.337 p < 0.001; β = 0.364 p = 0.001), but it did not act as a mediator of the effect on FA.

**Conclusion:**

Higher BMI is associated with right cingulate microstructure in BD, but not in CTR, and this effect could not be explained by inflammatory mediation alone.

## Introduction

Obesity is disquietingly common among individuals living with bipolar disorder (BD).^1 , 2^ Higher body mass index (BMI) is not only associated with increased cardiovascular risk,^3^ but is also associated with illness severity, with worse global functioning status, and with cognitive impairment,^4 , 5^ possibly through damage to neural substrates.^6^ Compromised white matter (WM) integrity estimated by fiber fractional anisotropy (FA) could be a candidate pathway for such deficits.^7 , 8^

The association between BMI and FA has been previously explored in the context of mood episodes in BD.^9 , 10^ During depression, BMI is associated with structural connectivity in cortico-limbic networks.^9^ Following the first episode of mania, the main findings are disruptions in right parietal, temporal, and occipital regions of overweight and obese patients.^10^ To our knowledge, there are no studies in euthymic patients. Although abnormalities during euthymia are less pronounced, they seem to reflect long-term and possibly irreversible structural damage and act as more stable markers in BD.^11^

Although the association between BMI and WM microstructure has been described previously,^9 , 10^ the neurobiological pathway linking this association with conditions remains unclear. The authors discussed a possible inflammatory process, but no inflammatory markers were actually assayed.^9 , 10^ C-reactive protein (CRP) is a sensitive marker of peripheral inflammation that has been extensively reported in obesity.^12^ Also, it has already been associated with WM microstructural damage in severe mental illness,^13^ so it constitutes a promising candidate to test the hypothesis of inflammatory damage in obese bipolar patients.

We hypothesize that obese individuals with BD in a euthymic phase present WM microstructural damage related to BMI, as a possible consequence of an unbalanced allostatic and pro-inflammatory profile.^4^ Therefore, this is a proof-of-concept study that aims to: 1) investigate associations between BMI and FA in individuals with BD during euthymia in comparison with a control group of healthy individuals (CTR) and 2) test whether any possible association is mediated by inflammation measured by CRP.

## Material and methods

This is a cross-sectional observational study that included 101 subjects, 35 BD and 66 CTR. To be included in the study, all participants needed to be adults (age > 18 years) at the time of enrollment and sign an informed consent form. The project was approved by the Research Ethics Committee at the Hospital de Clínicas de Porto Alegre (HCPA, Project ID 10-0348) and is in accordance with the Declaration of Helsinki.

BD patients diagnosed with type I BD receiving outpatient psychiatric care at the HCPA were eligible for enrollment on the study if euthymic. A trained psychiatrist confirmed the diagnosis using the structured clinical interview for DSM-IV (SCID), and euthymia was defined as Hamilton Rating Scale for Depression^14^ and Young Mania Rating Scale^15^ scores less than 7. Recruitment of CTR members followed the same logistics, selecting from among companions of individuals attending outpatient care of another medical specialty at the HCPA or community volunteers from the same socioeconomic background as the cases. For each CTR, the psychiatrist performed a clinical interview to rule out history of mental illness in the candidate or a first-degree relative.

The following exclusion criteria were applied for both groups: 1) age > 70 years; 2) pregnancy; 3) substance abuse or addiction (other than smoking); 3) endocrine or cardiovascular disease requiring medical attention or treatment adjustment; 4) rheumatological, neurological, autoimmune, infectious, or chronic inflammatory diseases; 5) immunosuppressive therapy; and 6) any contraindication precluding Magnetic Resonance Imaging (MRI) scanning.

Weight and height were obtained for each individual to calculate BMI (weight/height*height; kg/m^2^). The same instruments (scale and height ruler) were used for all participants, wearing light clothing but no shoes. A trained technician collected five milliliters of blood from each subject by venipuncture. High sensitivity CRP was measured using a latex particle-enhanced immunoturbidimetric assay following the manufacturer`s instructions (Roche Diagnostics, Indianapolis, IN, United States).

MRI images were acquired by a Philips Achieva 1.5T (Bethesda/Netherlands, 2009) with a dedicated 8 channel headcoil. Diffusion weighted MRI images were acquired using single-shot spin-echo echo-planar imaging (SE-EPI) sequence: TR/TE/Flip angle (10000 ms/124 ms/90º); b-value of 0 and 1000 s/mm^2^ with 15 directions; and voxel sizes: 2×2×3mm^3^ (high resolution). A trained researcher was responsible for processing and verifying all the volumetric segmentation using Freesurfer image analysis suite v.5.1.0, (http://surfer.nmr.mgh.harvard.edu/). TRACULA, a toolbox package within Freesurfer, was used for automated segmentation of major WM tracts.^16^

The *t* test for independent samples was used to assess differences between groups in age, BMI, and years of education, and the chi-square test was employed for sex and smoking status. Continuous variables are described as mean and standard deviation and categorical variables as percentages unless otherwise specified. Lithium use, age, and sex were included as covariates in all subsequent analyses because they are intimately related with brain microstructure.^17^ The Kolmogorov-Smirnov test was used to check the parametric distribution of the variables, including regression residuals. Because serum CRP levels were not normally distributed, we used the parametric distribution of logarithmic-transformed data for all analyses of this variable.

To avoid multiple comparisons problems, we applied a feature selection algorithm titled least absolute shrinkage and selection operator (LASSO) to select the most important regions of interest (ROI), using leaving-one-out cross validation (LOOCV). Afterwards, we ran Linear Regression Models for BMI main effect on FA ROI selected by LASSO and then conducted an analysis of mediation by serum CRP levels. The statistical program IBM SPSS Statistics 18.0 was used to compile data and conduct statistical analysis using regression models, while the statistical software R version 3.6.1 and the caret version 6.0 package were used for the feature selection algorithm.

## Results

The sample groups did not differ in age, smoking status, or years of education, as summarized in Table 1 . As expected, the average BMI was greater among patients with BD. Nineteen (54%) of the individuals with BD were taking lithium. Other medications in use by patients included valproate and atypical antipsychotics.

**Table 1:** Sociodemographic data of the sample across groups

	Individuals with bipolar disorder (n = 35)	Unaffected controls (n = 66)	Group comparisons
Age,* mean (SD)	42.35 (15.05)	37.58 (14.05)	t_(100)_ = -1.57, p = 0.119
Sex^†^ male/female, n (%)	11 (31)/24 (69)	38 (58)/28 (42)	χ^2^_(1)_ = 6.26, p = 0.012*
Smoking status,^†^ n (%)	8 (23)	8 (12)	χ^2^_(1)_ = 1.70, p = 0.192
Education years,* mean (SD)	9.94 (3.02)	10.88 (3.52)	t_(100)_ = 1.31, p = 0.193
BMI (kg/m^2^),* mean (SD)	29.70 (6.55)	25.54 (4.24)	t_(48.57)_ = -3.34, p = 0.002*
Obese individuals,^†^ n (%)	14 (40)	11 (16)	χ^2^_(1)_ = 6.26, p = 0.012*
YMRS, median (IR)	0.50 (3.00)	-	-
HAM-D, median (IR)	2.0 (5.00)	-	-
Lithium users, n (%)	19 (54)	-	-
Valproate users, n (%)	15 (42)	-	-
Atypical antipsychotic users, n (%)	22 (63)	-	-

BMI = body mass index; HAM-D = Hamilton Depression Rating Scale; IR = interquartile range; SD = standard deviation; YMRS = Young Mania Rating Scale.

* *t* test for independent variables.

^†^ Chi-square test.

The LASSO feature selection model consisted of 4 variables: sex (male as reference), left corticospinal tract FA, right cingulate gyrus endings FA, and forceps major of the corpus callosum FA with coefficients of -0.5, 0.16, -0.08, -0.06, respectively. The area under the curve was 0.62, sensitivity was 0.63, specificity was 0.62, and balanced accuracy was 0.63.

Each of the tracts that were identified in the LASSO model were considered ROI for further analyses and were entered into linear regression models. BMI and left corticospinal tract FA were positively associated in the overall sample (BD + CTR), controlled for covariates (AdjR^2^ = 0.319 F_(4)_ = 10.841 p < 0.001; β = 0.247 t = 2.460 p = 0.016).

**Figure 1:**
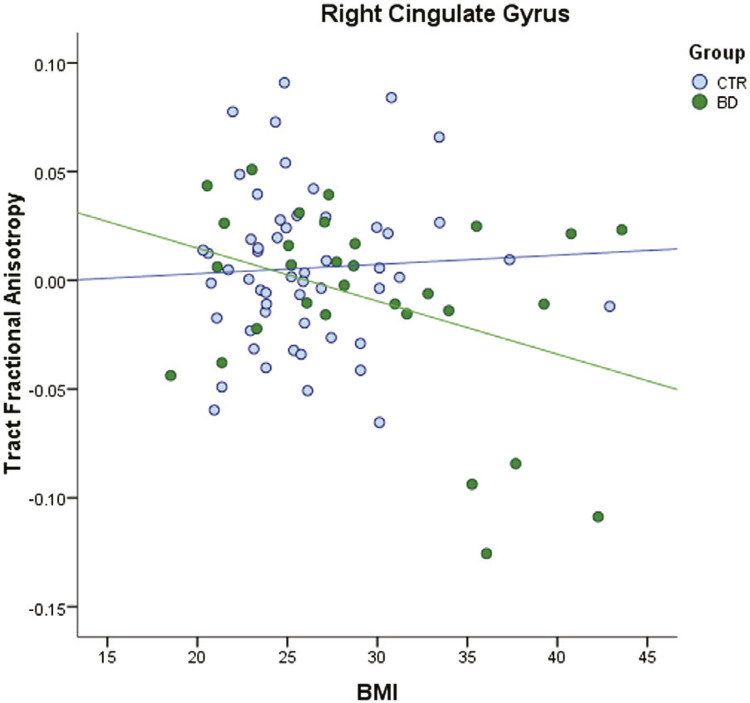
Regression analysis of BMI and FA of the right cingulate fibers across groups. Fractional anisotropy values shown are corrected for lithium use (for BD patients only), age, and sex. BD = bipolar disorder patients; BMI = body mass index (kg/m2); CTR = control group.

In the right cingulate gyrus, BMI showed a trend but did not reach statistical significance to predict the FA of the overall sample (AdjR^2^ = 0.139 F_(4)_ = 4.396 p = 0.003; β = -0.209 t = -1.855 p = 0.067). There was an interaction effect between BMI and diagnostic group (F_(5)_ = 3.5857 p = 0.012; F change = 0.227; AdjR^2^ = 0.093; BMI*Group effect: t = -2.011, β = -1.093, p = 0.048). This effect was not mediated by inflammation measured by CRP, since the direct effect of BMI*Group on right cingulate gyrus (c’= -0.0009 p = 0.0086) was greater than the total effect counting the indirect effect of CRP (c = -0.0008 p = 0.0203). In the BD subgroup analysis, BMI showed a negative correlation (AdjR^2^ = 0.312 F_(3)_ = 5.537 p = 0.004; β = -0.340 t = -2.235 p = 0.034), while in CTR there was no association ( Figure 1 ).

Fractional anisotropy values for the forceps major of the corpus callosum showed no significant correlation with BMI of the sample (AdjR^2^ = 0.088 F_(4)_ = 3.015 p = 0.023; β = 0.118 t = 1.014 p = 0.313).

In the overall sample, BMI was strongly correlated with higher levels of CRP (AdjR^2^ = 0.170 F_(3)_ = 7.337 p < 0.001; β = 0.364 t = 3.549 p = 0.001). In the analysis by group, this effect was prominent and probably driven by the CTR group (AdjR^2^ = 0.268 F_(3)_ = 8.327 p < 0.001; β = 0.418 t = 3.092 p = 0.003). However, there was no significant correlation among BD individuals, (AdjR^2^ = -0.003 F_(3)_ = 0.095 p = 0.422; β = 0.289 t = 1.622 p = 0.116).

## Discussion

The results of this study point to an association between BMI and decreased FA in the right cingulate gyrus of individuals with BD, but not in CTR. This association could indicate WM microstructural damage in this region.^18^ Decreased cingulate FA is one of the most consistent findings in BD.^18 , 19^ Loss of integrity in this structure is highly deleterious considering its central role of connection in the limbic system.^18^ Its posterior portion subserves an integrative network of cognitive tasks, top-down attentional control, visual processing, and memory systems for recognition,^20^ while the anterior portion is fundamental for the processing of executive functions related to emotional and visceromotor stimuli.^21^ Indeed, the cingulum links together regions critical for these processes, including the cingulate cortex, the ventral visual stream, and the hippocampal complex.^20^ In sum, decreased FA in association fibers such as the cingulate gyrus provide evidence of WM dysconnectivity in BD,^17^ and our findings suggest that it may be associated with BMI.

Accelerated aging of the WM, especially in limbic communication structures, could be the pathway underlying such microstructural damage associated with BMI in BD, but with uncertain etiology.^7^ Chronic inflammatory status is a good candidate for the neuroimmunological abnormalities that occur in severe psychiatric disorders^8^ and could mediate the deleterious effect of BMI in WM.^9 , 10^ In our study, higher BMI was indeed correlated with increased pro-inflammatory cytokines in our population measured by CRP, but this correlation was not seen in BD, only among CTR.

In contrast to what we had expected, the decreased right cingulate fiber FA in BD was not mediated by CRP. This suggests that a metabolic process other than inflammation may be playing a role in the structures we studied, conferring microstructural damage or protection related to changes in BMI. Cholesterol, triglycerides, and glucose levels have also been correlated with FA structures in BD.^9^ Insulin resistance plays a prominent role in obese BD, modulating white matter abnormalities and synaptic plasticity alongside inflammatory dysfunction and oxidative stress.^4^ Furthermore, adipose tissue hormonal secretion and behavioral challenges such as food intake and exercise balance have been consistently reported among obese BD patients^2 , 3^ and could be mediating WM microstructural damage.^6^

One possible explanation for the lack of mediation effect of CRP on the FA of cingulate fibers is that our BD sample was out of episode during the assessment, when inflammation may decrease.^8 , 11^ Our hypothesis was that even in recovery obese patients would present higher inflammatory status, which was not confirmed.

We also found a positive association between BMI and the left corticospinal tract among both BD and CTR. In contrast to right cingulate fibers, corticospinal tracts are reported to have higher FA in BD than in the general population.^22^ Higher FA in this essentially motor tract could perpetuate deficits in motor inhibition,^17^ which in turn can contribute to behavioral features observed in obese BD as they struggle with inhibition of exaggerated physical and emotional responses.^23^

There are important limitations to address in the present study. The lack of association with BMI in CTR should be interpreted with caution, because of limited variability of BMI among this CTR sample. Compromised WM has been described in larger obese non-psychiatric populations.^7^ The cross-sectional design of this study does not allow inferences about causality and this limitation cannot be adequately mitigated without a longitudinal follow-up. The sample size was modest, which limited the possibility of including more covariates and performing sensitivity analyses. Also, patients were on continuous use of medications that might influence the integrity of the neural bundles,^24^ such as anticonvulsants and antipsychotics, which we could not control for because of the small sample size. Nonetheless, we were able to control for lithium use.

In summary, we found that BMI was associated with WM microstructure in euthymic bipolar patients. Such results reinforce the hypothesis that there are convergent pathways between BD and systemic alterations associated with obesity, contributing to the understanding of both conditions and their bilateral relations. Furthermore, our findings do not corroborate an inflammatory pathophysiology underlying this association and future efforts in this field could include the endocrine profile beyond inflammatory markers.
